# Do Patients with Multiple Sclerosis Derive More Benefit from Robot-Assisted Gait Training Compared with Conventional Walking Therapy on Motor Function? A Meta-analysis

**DOI:** 10.3389/fneur.2017.00260

**Published:** 2017-06-13

**Authors:** Xiao Xie, Hao Sun, Qing Zeng, Pengcheng Lu, Yijin Zhao, Tao Fan, Guozhi Huang

**Affiliations:** ^1^Department of Rehabilitation, Zhujiang Hospital, Southern Medical University, Guangzhou, China; ^2^The Guangdong General Hospital, Guangzhou, China

**Keywords:** multiple sclerosis, robot-assisted gait training, gait, rehabilitation, meta-analysis

## Abstract

**Objective:**

To determine whether robot-assisted gait training (RAGT) is more effective in improving mobility, endurance, gait performance, and balance in patients with multiple sclerosis (MS) compared with conventional walking rehabilitation treatment (CWT).

**Data sources:**

Sources included the Cochrane Library, PubMed, Embase, and Science Direct databases.

**Review method:**

All possible articles were retrieved by two independent investigators and relevant articles were gathered. Studies on adult patients (older than 19 years old) suffering from MS were included, regardless the subtype of MS diagnosis. Finally, we identified seven studies that comprised 205 patients with MS.

**Results:**

We identified seven studies comprising 205 patients with MS in our meta-analysis. The pooled mean difference (MD) for the six-minute walk test (6MWT) was 14.25 [95% confidence interval (CI) 3.19 to 25.32, *Z* = 2.53, *P* = 0.01, *I*^2^ = 54%], which indicates that RAGT is superior to CWT on improving endurance. No significant improvement on using RAGT was found regarding the Berg Balance Scale (MD = −0.59, 95% CI: −2.7 to 1.52, *Z* = 0.55, *P* = 0.58, *I*^2^ = 51%), 10-meter walk test [standard mean difference (SMD) = 0.03, 95% CI: −0.26 to 0.31, *Z* = 0.18, *P* = 0.86, *I*^2^ = 48%] timed up and go (TUG) test (MD = −1.04, 95% CI: −8.68 to 6.60, *Z* = 0.27, *P* = 0.79), or stride length (SMD = 0.36, 95% CI: −0.13 to 0.85, *Z* = 0.73, *P* = 0.15).

**Conclusion:**

We can conclude that RAGT can bring more benefits on improving 6MWT among MS patients, but it is not enough to make a clinically significance conclusion. Considering the limitation of our study, it takes reservations about recommending all MS patients to take RAGT as primary rehabilitation intervention. Unless patients with progressive MS can take conventional rehabilitation in early time, RAGT would be a suitable substitute.

## Introduction

Approximately 75% of multiple sclerosis (MS) patients experience mobility problems. Gait rehabilitation, which can increase patients’ levels of activity and participation, thus increasing their independence, is of utmost importance (1). Physiotherapy has been shown to be effective for improving gait function compared with no treatment among MS patients (2).

However, traditional over-ground walking training is associated with a high fall risk during treatment for patients with more severe gait disabilities and cannot be conducted in the early stage of rehabilitation (3). An alternative treatment for these patients is body-weight supported treadmill training (BWSTT), which enables early initiation of gait training and has been shown to be effective in patients who have had a stroke (4–6). However, among MS patients, few studies have found BWSTT to be more effective than the traditional over-ground walking training. The manual administration of BWSTT is difficult and greatly depends on the physiotherapist’s abilities, thus making BWSTT inefficient (7, 8).

To facilitate the delivery of BWSTT, robot-assisted gait training (RAGT) was recently developed, and it has many advantages over BWSTT and conventional walking treatment. The RAGT is reproducible, stable, more physiological, and measurable (9). However, series of clinical trials tried to verify whether RAGT is more effective or not, but failed to gain positive results. On one hand, the small number of MS patients treated by RAGT in these studies lead to low statistical power. On the other hand, trails and animal test have proved that Lokomat has limitations in precisely replicating a normal gait, as it restricts the movement of the trunk and pelvis and induces a different muscle activation pattern for the lower limbs. Although its results are based solely on the kinematic pattern which was adjusted according to the feeling of the patients. And only animal experiment was conducted to verify variable training paradigms appears to be a more effective rehabilitative strategy than the fixed training paradigm. So, the question remains open with regard to whether RAGT offers better outcome than regular physiotherapy (10).

To date, no meta-analysis has been conducted regarding gait function rehabilitation by RAGT for patients with MS. The objective of this study was to determine whether people suffering from MS could benefit more from RAGT compared with conventional walking rehabilitation treatment (CWT) on motor function.

## Materials and Methods

### Literature Search

A comprehensive search was conducted in the Cochrane Library, Science Direct, and PubMed databases prior to 2016. Two reviewers (Xie X and Sun H) independently searched articles in electronic databases above using the search strategy “(((((MS) OR disseminated sclerosis) OR encephalomyelitis disseminate)) AND ((((((RAGT) OR robot-assisted gait rehabilitation) OR robotic-assisted gait rehabilitation) OR robotic walking therapy) OR robotic locomotor training) OR robotic-assisted locomotor training)) AND (((CWT) OR conventional physical therapy) OR Conventional therapy).” Reference lists from related articles were also reviewed. The language was restricted to English. All possible articles were retrieved by two independent investigators and relevant articles were gathered. The search strategy was also presented as a Data Sheet S1 in Supplementary Material.

### Type of Studies

Studies on adult patients (over 19 years old) suffering from MS were included, regardless the subtype of MS diagnosis, as were effect studies on RAGT that encompassed improving gait function and gait-related outcome measurements. Studies were also included if at least one of the intervention groups received RAGT exclusively as an intervention. Studies where RAGT was used in combination with interventions other than BWS were excluded. For example, if RAGT was combined with functional electrostimulation, the study was excluded. Studies with outcomes focused exclusively on physical capacity, electromyography or kinematic data, and/or cardiorespiratory functioning were excluded. Animal studies and studies on children also were excluded. Pre-, quasi-, and true-experimental studies were included.

### Study Selection and Data Extraction

All studies included met the following criteria:

1. Must be random clinical trials (included pilot random clinical trials).
2. Language restriction was not applied, and the nationality and race of research subjects were not restricted.
3. Trials included must compare the RAGT and CWT.
4. The primary outcome is six-minute walk test (6MWT), 10MWD, Berg Balance Scale (BBS) and the secondary outcome is some other gait parameters [e.g., stride length (SL)].

Studies containing the following criteria have been excluded:

1. The research failed to provide the key information, such as the total number of patients.
2. Studies were excluded if RAGT was used in combination with interventions other than BWSTT (body-weight support treadmill training).
3. Studies were excluded if outcomes focused exclusively on physical capacity, electromyography or kinematic data, and/or cardiorespiratory functioning. Animal studies and studies on children also were excluded.

### Type of Outcomes

Improving activity and participation is as important as reducing impairments among patients suffering from MS. The walking speed is a performance measure used to evaluate functional mobility, gait, and vestibular function. The outcome of the walking speed has been shown to have excellent correlation with dependence in self-care and domestic life among patients with MS (11). 6MWT was used as a performance-based measure of functional exercise capacity. The BBS is a 14-item objective measure designed to assess static balance and fall risk in adult populations. This measure is highly recommended by the American Physical Therapy Association’s MS Taskforce (MSEDGE) to be conducted among patients suffering from MS. Other outcomes are used as secondary outcomes.

1. Primary outcomes:
  (1) Mobility [e.g., 6MWT, 20- or 10-meter walk test (10MWT), gait speed]
  (2) Gait performance [e.g., timed up and go test (TUG)]
  (3) Balance (BBS)
2. Secondary outcomes:
  (1) Gait parameters (e.g., SL)

### Statistical Analysis

Means and SD were determined for each outcome in each treatment group. For the combined data weighted mean difference, mean difference (MD) and 95% confidence interval (CI) were computed. Review Manager 5.3 software (The Nordic Cochrane Center, The Cochrane Collaboration, Copenhagen, Denmark) was used for all analyses. The results of the meta-analysis are presented using forest plots. The heterogeneity of the included studies was quantified by the *I*^2^ statistic, which indicates the percentage of variation across studies due to heterogeneity. A fixed effects model was used to combine studies when the *I*^2^ value was less than 60%. Otherwise, the random effects model was used (12).

Bias risks were assessed according to the criteria outlined in the Cochrane Handbook for Systematic Reviews of Interventions by two independent authors (12). Disagreements were resolved through consensus. Publication bias was not assessed because there were only seven studies in the meta-analysis, and the test power would have been too low to distinguish change from real asymmetry (13).

## Results

**Figure 1:**
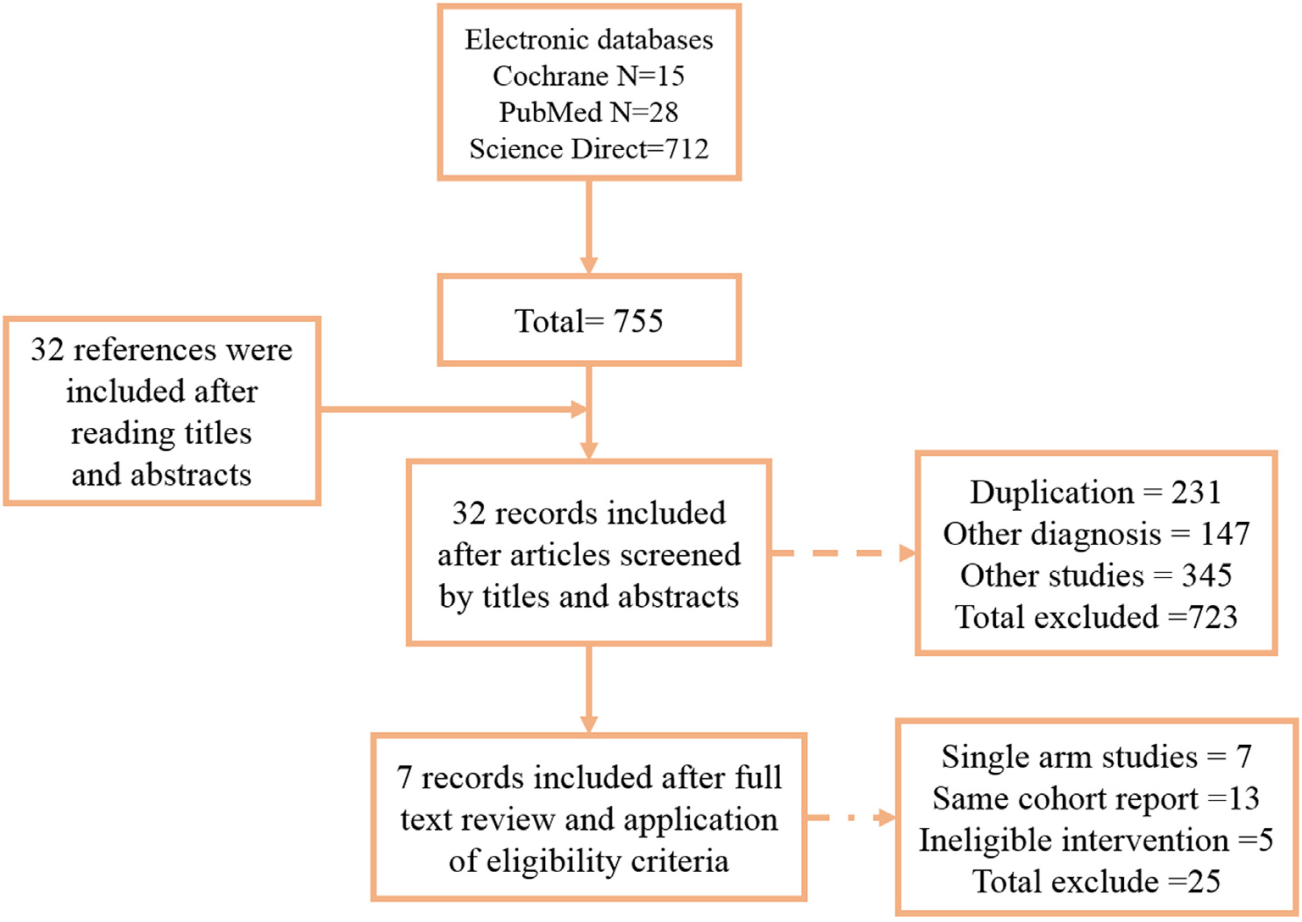
Flow diagram. Seven articles were included in this meta-analysis.

According to the inclusion criteria, we identified seven studies that comprised 205 patients with MS (14–19). The flow diagram of the study selection is presented in Figure 1. All seven studies were conducted with random sequence generation, the quality evaluations of each clinical trials are listed in Table 2. The risk of bias in the included studies was assessed using the standard Cochrane Collaboration tool. All of the included randomized controlled trials (RCTs) with straight random principles were suggested to have a low risk, despite the lack of double-blinding, so the assessments were considered to be non-biased. The individual prognostic factors (e.g., age, gender, and performance status) were all well-balanced within these studies.

Among all included studies, Sofia and Beer et al. (18, 20) limit the inclusion criteria to severe gait impairments as evidenced by a rating on the Expanded Disability Status Scale (EDSS) between 6.0 and 7.0. All studies set Lokomat as the treatment of RAGT group except Gandolfi et al. (14) (electromechanical Gait Trainer). The therapy sessions differ from 9 to 12. All the patient characteristics, such as gender, EDSS score, and age, are summarized in Table 1. Different protocols of reporting among the included studies are also reported in Table 1. In our analysis, four studies measured walking speed by the 10MWT. Walking endurance was measured with the 6MWT in three studies. Balance function rehabilitation among patients was evaluated by the BBS in three RCTs. Other gait parameters, including gait speed, cadence, double support time (DST), and SL, were reported in different studies. Straudi et al. (15) and Gandolfi et al. (14) also used the GAIT-Rite mat to measure several of the gait parameters noted above.

**Table 1:** The characteristics of all identified studies.

Reference	Participants				Intervention(+comparison)		Outcome	
	Num	Male/female	Expanded Disability Status Scale (EDSS) score	Age	Intervention	Comparison	Outcome measurements	Assessment times
Lo and Triche (19)	13	7 m/6 f robot-assisted gait training (RAGT) 3 m/3 f B: 4 m/3 f	RAGT: 5.0 (1.6); body-weight supported treadmill training (BWSTT) 4.9 (0.9)	RAGT 50.2 (11.4); BWSTT 49.6 (11.8)	RAGT: BWSTT + Lokomat (*n* = 6), 2 sessions/week for 6 weeks	BWSTT (*n* = 7), 2 sessions/week for 6 weeks	25-foot walk (T25FW), 6-min walk double support time (DST)	Baseline Phase I

Beer et al. (18)	29 (35: 6 dropouts; 5 in RATT and 1 in CWT)	12 m/23 f RAGT: 7 m/12 f, CWT: 5 m/11 f	RAGT: 6.5 (range: 6–7.5); CWT: 6.5 (range: 6–7.5)	RAGT: 49.7 (SD 11); CWT: 51 (SD 15.5)	RAGT: BWSTT + Lokomat (*n* = 14), 5 sessions/week for 3 weeks	CWT: conventional walking train (*n* = 15), 5 sessions/week for 3 weeks	Walking speed (20MWT), walking endurance (6MWT), stride length (SL) (cm)	Baseline, after 3 weeks and at follow-up after 6 month

Gijbels et al. (22)	49 (67: 18 dropouts)	NG	RAGT: 5.9 (range: 3–6.5); CWT: 5.7 (range: 3–6.5)	RAGT: 58.2 (range: 37–73); CWT: 54.2 (range: 36–74)	RAGT: BWSTT + Lokomat (*n* = 26), 30 min/session9 session	CWT: walking in group with physiotherapist (*n* = 23), 30 min/session 9 session	Walking speed (10MWT, 3MWT on 80 m hallway)	Baseline, after treatment

Schwartz et al. (17)	28 (32: 4 drop outs; 1 in CWT, 3 in RAGT)	14 m/18 f RAGT: 7 m/8 f, CWT: 7 m/10 f	RAGT: 6.2 (range: 5.5–7); CWT: 6 (range: 5–7)	RAGT: 46.8 (range: 29–69); CWT: 50.5 (range: 28–70)	RAGT: BWSTT + Lokomat (*n* = 15), 2–3 sessions/week for 6 weeks	CWT: Gait and dynamic balance exercises (*n* = 17), 2–3 sessions/week for 6 weeks	Walking speed (10MWT), walking endurance (6MWT), disability (EDSS)	Baseline, after 4 weeks, follow-up after 3 and 6 months

Straudi et al. (15)	16 (18: 2 drop outs; 1 in CWT, 3 in RAGT)	5 m/11 f RAGT 5 m/5 f CWT 1 m/7 f	RAGT 5.8 ± 0.8; CWT 5.7 ± 0.7	RAGT: 49.6 ± 12.0; CWT: 61.0 ± 8.8	RAGT: Lokomat (*n* = 8), 2 sessions/week for 6 weeks	CWT: lower-limb and lower-limb muscles exercises (*n* = 8), 2 sessions/week for 6 weeks	Six-minute walk test (6MWT) timed up and go test (TUG) gait speed, cadence, double support, step length, step time	Week prior to treatment initiation (T0), the week after the end of treatment (T1) and at 3 months’ follow-up (T2)

Gandolfi et al. (14)	24 (26: 2 drop outs.2 in RAGT)	6 m/16 f RAGT 5 m/7 f SIBT 1 m/9 f	RAGT 3.96 (0.75); SIBT 4.35 (0.67)	RAGT 50.83 (8.42); SIBT 50.1 (6.29)	RAGT: electromechanical Gait Trainer GT1 (Reha-Stim, Berlin, Germany) (*n* = 12), 2–3 sessions/week for 6 weeks	SIBT: sensory integration balance training (*n* = 12), 2–3 sessions/week for 6 weeks	Gait speed (cm/s) Berg Balance Scale (BBS) GAITRite System: gait speed, cadence, double support, step length, step time	Baseline (T0), after treatment (T1) and at 1-month follow-up (T2)

Straudi et al. (20)	58	18 m/34 f RAGT 10/17 CWT 8/7	RAGT 6.43 (0.38); CWT 6.46 (0.43)	RAGT 52.26 (11.11); CWT 54.12 (11.44)	RAGT (Lokomat) *N* = 27, 2–3 sessions/week for 6 weeks	CWT *N* = 25, 2–3 sessions/week for 6 weeks	10MWV/6MWT/BBS/TUG/SL/SF	Baseline, after 4 week, follow-up after 3 and 6 months

**Table 2:** The risk bias of included studies.

Risk domains	Lo and Triche (19)	Beer et al. (18)	Vaney et al. (16)	Schwartz et al. (17)	Straudi et al. (15)	Gandolfi et al. (14)	Straudi et al. (20)
Random sequence generation	Yes	Yes	Yes	Yes	Yes	Yes	Yes
Allocation concealment	Yes	Yes	Yes	Yes	Yes	Yes	Yes
Blinding of participants and personnel	No	No	Yes	Yes	No	No	Yes
Blinding of outcome assessment	No	No	No	No	No	No	No
Incomplete outcome data	No	No	No	No	No	No	No
Selective reporting	No	No	No	No	No	No	No

### Walking Speed

**Figure 2:**
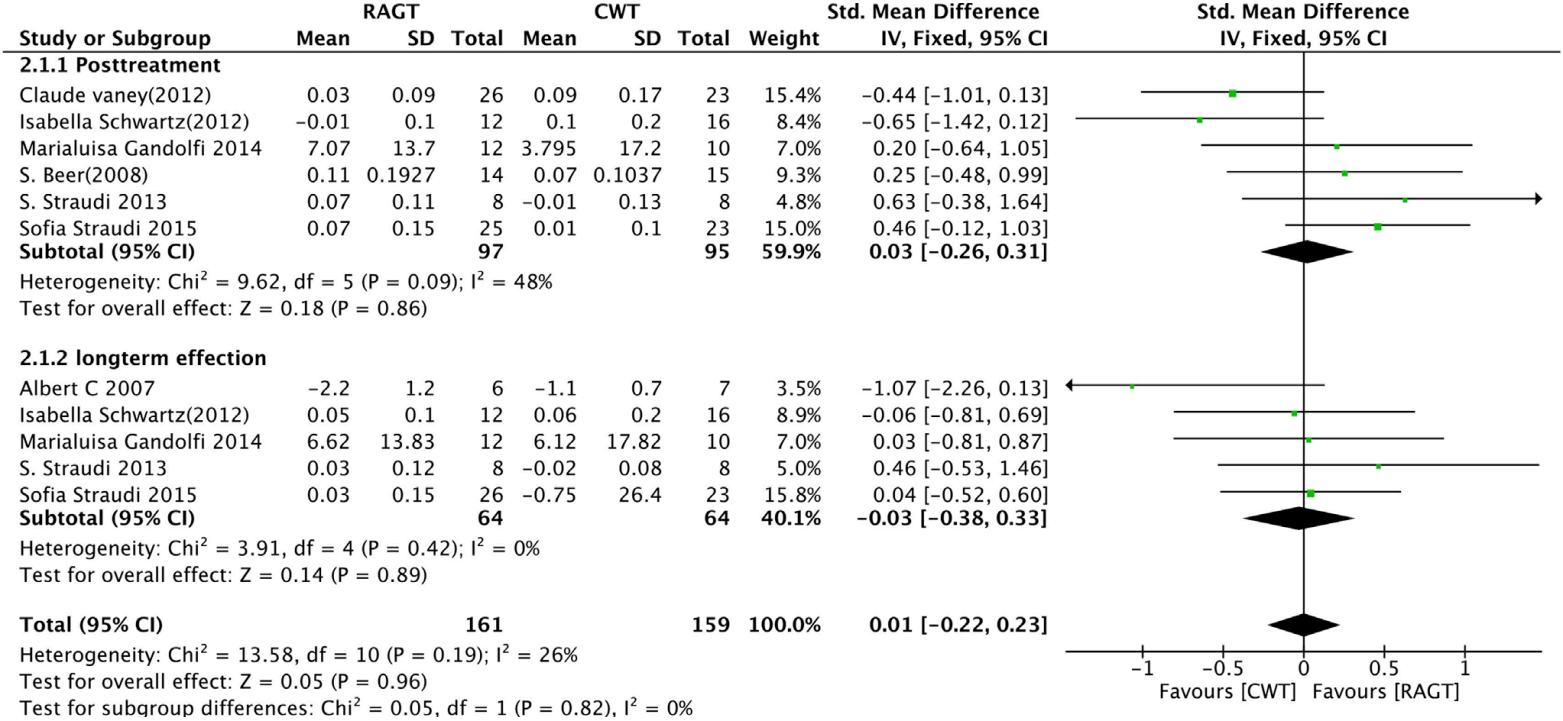
Forest plot of comparison for the walking speed. Robot-assisted gait training (RAGT) versus CWT for walking speed among patients with multiple sclerosis (top, post-treatment; bottom, follow-up data included).

Four studies measured walking speed using 10MWT, and two study using gait speed directly. All raw data are listed in Data Sheet S1 in Supplementary Material. The pooled standard mean difference (SMD) for the walking speed was 0.03 (95% CI: −0.26 to 0.31, *Z* = 0.18, *P* = 0.86, *I*^2^ = 48%), no significant differences were observed between RAGT and the control group (Figure 2).

### Endurance

**Figure 3:**
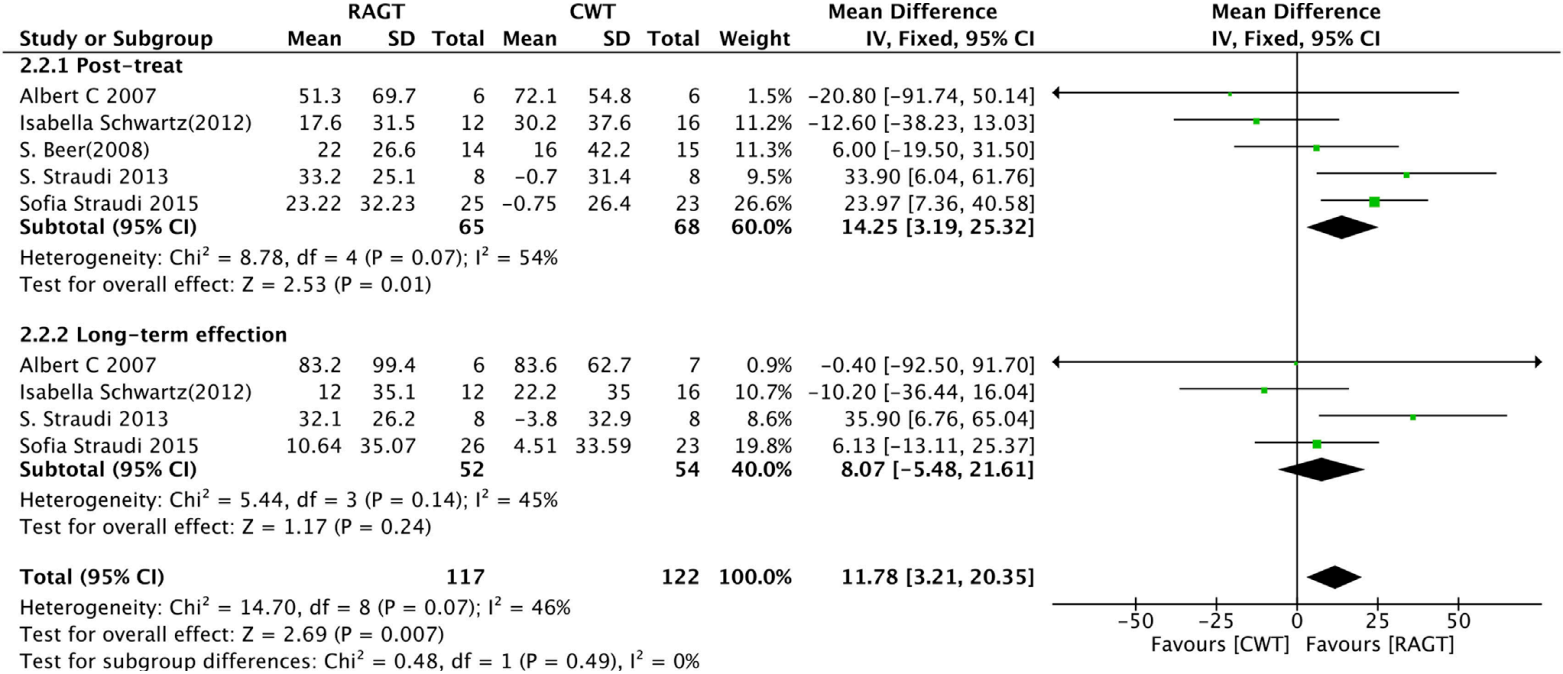
Forest plot of comparison for the six-minute walk test. Robot-assisted gait training (RAGT) versus CWT for exercise tolerance among patients with multiple sclerosis (top, post-treatment; bottom, follow-up data included).

Five studies measured exercise endurance using the 6MWT. Because all of the baseline data included are in balance, we used the raw data directly to compute the pooled MD, which resulted MD = 14.25 (95% CI: 3.19 to 25.32, *Z* = 2.53, *P* = 0.01, *I*^2^ = 54%), indicating that RAGT is statistically superior to CWT on improving endurance (Figure 3). Four trials (15, 17) also reported follow-up data, so we performed a sensitivity analysis based on the different time points (MD 8.07, 95% CI: −5.48 to 21.61, *Z* = 1.17, *P* = 0.21, *I*^2^ = 45%). However, there is no significant difference between RAGT and CWT about long-term effect for improving endurance (Figure 3).

### Balance

**Figure 4:**
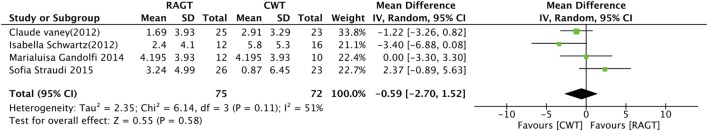
Forest plot of comparison for the Berg Balance Scale. Robot-assisted gait training (RAGT) versus CWT for balance function among patients with multiple sclerosis.

Four trials reported the outcome of the BBS in MS subjects. The pooled MD was −0.59 (95% CI: −2.7 to 1.52, *Z* = 0.55, *P* = 0.58, *I*^2^ = 51%). No clinically significant difference was observed (Figure 4). It suggests that RAGT cannot bring more benefits on improving static balance among MS patients.

### Gait Performance and Gait Parameters

#### Timed Up and Go Test

**Figure 5:**
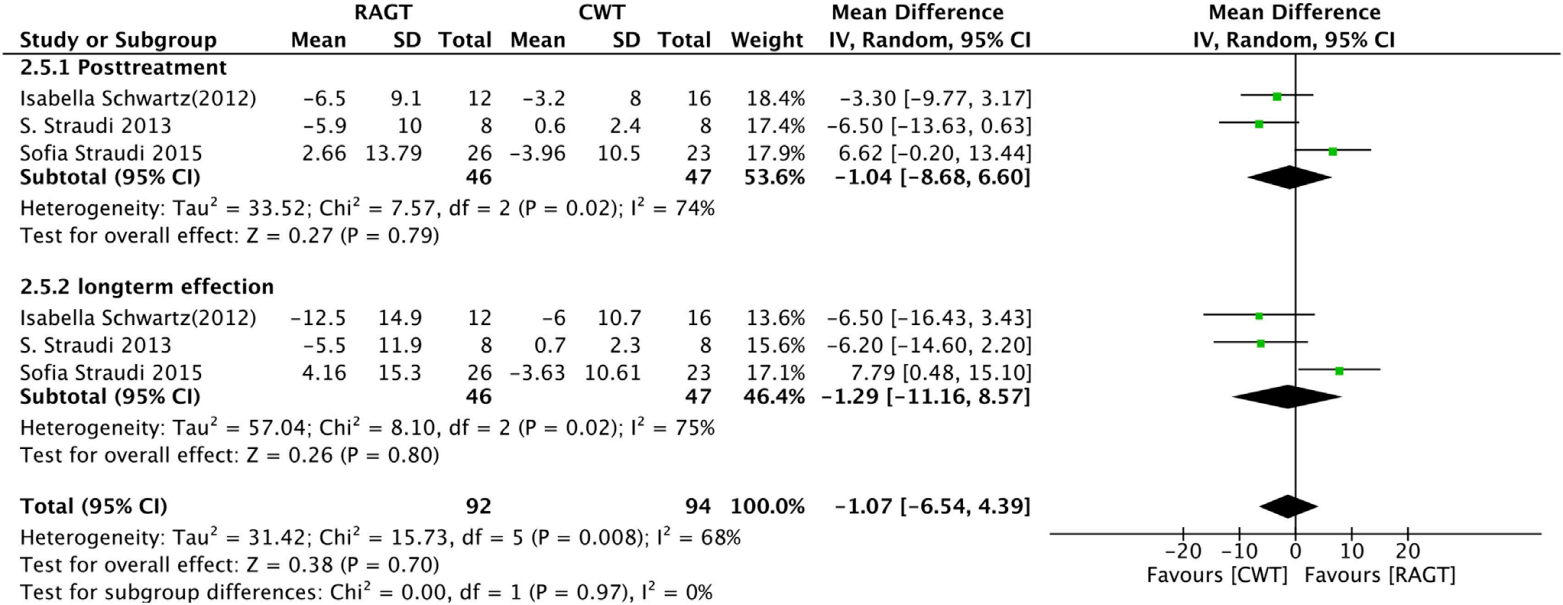
Forest plot of comparison for the timed up and go test. Robot-assisted gait training (RAGT) versus CWT among patients with multiple sclerosis (top, post-treatment; bottom, follow-up data included).

We summarized the results for the effects of RAGT on gait performance as measured by the TUG, which is used to assess mobility, balance, walking ability, and fall risk in older adults. Both post-intervention and follow-up data from three studies were analyzed. However, no significant differences were observed between the RAGT group and the control group regarding TUG (post-intervention: MD = −1.04, 95% CI: −8.68 to 6.60, *Z* = 0.27, *P* = 0.79; follow-up: MD = −1.29, 95% CI: −11.16 to 8.57, *Z* = 0.26, *P* = 0.80) (Figure 5).

#### Stride Length

**Figure 6:**
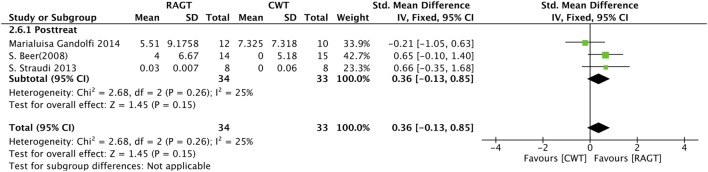
Forest plot of comparison for stride length. Robot-assisted gait training (RAGT) versus CWT among patients with multiple sclerosis.

Stride length was reported in three studies that were used to calculate the pooled SMDs. No significant difference was observed between the RAGT group and the control group (SMD = 0.36, 95% CI: −0.13 to 0.85, *Z* = 0.73, *P* = 0.15) (Figure 6).

## Discussion

Recommendations from MSEDGE (Neurology Section of the American Physical Therapy Association’s Multiple Sclerosis Taskforce) suggest that the increase in walking distance of 6MWT is correlated with better self-care, mobility, and domestic life. Several studies also showed that the walking capacity tests (6MWT or 2MWT eta) are highly correlated with habitual walking performance in MS.

Estimating the SD of changes from baseline may cause an imprecise result, so we use the difference between baseline and post-intervention to calculate the MD or SMD for a higher degree of certainty. Based on the latest data up to 2016, this meta-analysis suggests that RAGT can derive more benefit for patients with MS in improving endurance to some extent. But it seems that it is not enough to make a clinically significant conclusion based on the result. We found an improvement of 14.25 m in RAGT group, less than the minimally important change (22 m) for walking improvement from the patient perspective (21). Although this meta-analysis conducts a significant statistical difference (*P*-value ≤ 0.05) in the 6MWT among groups, it is too rash to demonstrate that RAGT can bring significantly clinical benefits so far.

Ten-meter walk test, which can assess the short duration walking speed. It has been proved to be a responsive test well suited to evaluate clinical interventions in various patient populations including stroke, Parkinson’s disease, general neurologic movement disorders. But our analysis did not reveal any statistically significant differences in the 10MWT between RAGT and control group. So, it suggests that RAGT cannot bring more benefit for patients in improving walking speed during short-distance walking. One possible explanation is that long-distance, rather short-distance, tests are more suitable in detecting improvements after rehabilitation for MS patients (22, 23).

On the other hand, it also has been reported that different types of walking training lead to differences in biomechanics and physiology (10, 24–26). One clinical concern that is often raised with Lokomat gait training is the lack of variability in the gait patterns. As RAGT is often run with 100% guidance force, patients are kept in a particular gait pattern, regardless of the subject’s intentions. Consequently, the participants lack the ability to vary their kinematic patterns from step to step. The ability to adjust gait pattern plays an important role during dynamic walking procedures. This finding may explain why RAGT cannot bring more effective motor function rehabilitation compared to CWT among MS patients, according to the outcome of the 10MWV. As only Lokomat restricting free trunk movements among RAGTs, analyze whether the obtained effects on walking endurance and balance are different in studies with Lokomat versus other robots which do not restrict the trunk is necessary. But only one study (14) included in our analyses used electromechanical Gait Trainer (Reha-Stim, Berlin, Germany) other than Lokomat, we cannot conduct a subgroup analysis due to the limit data. Further studies should aim at adjusting the working pattern of Lokomat and gain more improvements in precisely replicating a normal gait.

For functional balance tests, the BBS is generally considered the gold standard (27). The BBS is also an ideal measurement to evaluate the benefit of an intervention as a response to change. Only four trials have been published which evaluated balance function using the BBS in patients with MS. Our study was unable to provide any suggestions for clinical practice regarding which is the most effective treatment to improve balance function for MS patients. Based on our results, we suggest the effectiveness of RAGT on balance rehabilitation is approximately equal to CWT.

Due to the limited data, we did not detect any meaningful clinical results regarding the other parameters, such as SL and TUG. The pooled SMD calculated from each outcome did not reveal any differences between the RAGT and CWT group. The insufficient number of trials made it difficult to achieve a positive result. Moreover, the limited number of participants in each trial also made the analysis difficult. The limited number of participants is likely because the selection of patients for intervention studies is challenging, especially with the MS population, due to the variability in symptoms, the different types of MS, and the different and unpredictable courses of the disease (1).

Only two included clinical trials (18, 20) tested the effects of RAGT and compared it to conventional physiotherapy in patients with progressive MS and severe gait disability (EDSS 6.0–7.0). As it accounts for a large weighting (63.1%) in our analysis, we exclude these studies and make a new pooled analysis to verify whether there are still more benefits in RAGT group among patients with moderate MS (EDSS 6.0–7.0). The standard pooled MD for endurance was 0.04 (95% CI: −0.51 to 0.58, *Z* = 0.13, *P* = 0.89, *I*^2^ = 62%). There is no significant difference between the RAGT and CWT. Considering this limitation of our study, it takes reservations about recommending all MS patients to take RAGT as primary rehabilitation intervention. Unless patients with progressive MS can take conventional rehabilitation in early time, RAGT can be a suitable substitute.

### Study Limitations

There are several limitations in our study. First, only one included clinical trials tested the effects of RAGT and compared it to conventional physiotherapy in patients with progressive MS and severe gait disability (EDSS 6.0–7.0), so we cannot conduct a subgroup analysis to make a more precise recommendation about the most suitable patients for RAGT. Second, the CWT group also included body-weight support gait training. With the limited data, we could not conduct a subgroup analysis to compare this training with other gait retaliation treatments, such as ground walking training. Third, the small number of included RCTs made the subgroup and sensitivity analysis difficult. Therefore, we did not find any clinically significant differences except for the 6MWT. Fourth, we restricted the language of the studies to English, which may have caused us to miss relevant studies published in other languages.

## Conclusion

We can conclude that RAGT can bring more benefits on improving 6MWT among MS patients, but it is not enough to make a clinical significance conclusion. We take reservations about recommending all MS patients to take RAGT as primary rehabilitation intervention. With a limited amount of literature related to RAGT in people with MS, there is no significant difference in ameliorating walking speed and functional balance between RAGT and CWT. More RCTs with larger, but more homogeneous, populations are needed to conduct subgroup analysis for more precise clinical advice.

## Author Contributions

Study concepts and study design: GH and HS. Literature research and data acquisition: XX, QZ, PL, and YZ. Data analysis/interpretation and statistical analysis: HS. Manuscript preparation: XX. Manuscript editing: HS and XX. XX and HS contributed equally to this work and should be considered co-first authors.

## Conflict of Interest Statement

The authors declare that the research was conducted in the absence of any commercial or financial relationships that could be construed as a potential conflict of interest.

## Abbreviations

MS, multiple sclerosis; BWSTT, body-weight supported treadmill training; RAGT, robot-assisted gait training; CWT, conventional walking rehabilitation treatment; RCTs, randomized controlled trials; 10MWV, ten-meter walk velocity; 6MWT, six-minute walk test; BBS, Berg Balance Scale; TUG, timed up and go test; SL, stride length.
